# Arrhythmias and Cognitive Function: What is the Best Practice?

**DOI:** 10.19102/icrm.2018.091207

**Published:** 2018-12-15

**Authors:** Akash Rusia, Rahul N. Doshi

**Affiliations:** ^1^Division of Cardiology, Keck School of Medicine, University of Southern California, Los Angeles, CA, USA

**Keywords:** Ablation, atrial fibrillation, cerebrovascular accident, cognitive function, dementia

With the release of the 2018 European Heart Rhythm Association (EHRA)/Heart Rhythm Society (HRS)/Asia-Pacific Heart Rhythm Society (APHRS)/Latin American Heart Rhythm Society (LAHRS) expert consensus on arrhythmias and cognitive function,^1^ electrophysiology has received its first set of recommendations on how to address a problem that has become increasingly studied in the last decade: cognitive decline in patients with arrhythmias. The consensus statement reviews data showing an association between cognitive dysfunction and a variety of arrhythmias, cardiac arrest, and electrophysiology procedures. The authors provide scientific recommendations according to European Society Cardiology guidelines (categorized into “should do this,” “may do this,” and “do not do this” for ease of understanding). Here, we will discuss the significance of the consensus statement for considering cognitive decline in arrhythmia management and how these new recommendations might affect clinical practice.

## Atrial fibrillation

Although atrial fibrillation (AF) has long been known to be a major risk for stroke (which in itself is a major risk factor for dementia^2^), the most compelling point that the aforementioned consensus statement makes is when discussing the evidence of dementia in AF patients who have no clinical history or even an instance of silent stroke. In 2012, Santangeli et al. showed that AF is an independent risk factor for dementia in a meta-analysis of more than 77,000 patients.^3^ This study demonstrated a hazard ratio of 1.42 for AF patients without stroke developing dementia, which is comparable to the findings of other studies but is much lower than the relative risk of 2.7 for patients with both AF and a history of stroke.^4^ A follow-up study in 2013 by Thacker et al. showed cognitive function test scores declined faster in patients with AF versus in patients without AF, independently of stroke history.^5^ Although there appears to be solid evidence of an association between AF and both vascular dementia and Alzheimer’s disease in the absence of stroke, the focus of most practitioners should be on the implications in clinical practice.

Given that stroke prevention with anticoagulation is a known commodity, there is a new focus on the prevention of cognitive decline in AF patients without stroke. An enlightening study from 2014 by Jacobs et al. illustrated that, for patients on vitamin K antagonists (VKAs), the time in therapeutic range was inversely associated with the risk of dementia.^6^ The risk of dementia was increased not only by the time in subtherapeutic range but also by the time in supratherapeutic range, giving credence to the idea that microemboli and microhemorrhages may contribute to the development of dementia in the long-term. Knowing that dose titration is less of an issue for patients on novel oral anticoagulants (NOACs), there is a new focus on conducting studies comparing NOACs and VKAs for the prevention of dementia in patients with nonvalvular AF. Two ongoing clinical trials listed at ClinicalTrials.gov (NCT01994265 and NCT03061006) plan to compare warfarin and dabigatran with an endpoint of cognitive impairment and dementia.

The guidelines give a “may do this” recommendation for using NOACs instead of VKAs for stroke prevention in AF, with the rationale that it may have a beneficial effect on cognitive disorders. The authors detail a similar strength recommendation for performing cognitive assessments in AF patients with a suspicion of cognitive impairment. Ultimately, the message appears to be that help is on the way in the form of a number of clinical trials, with the hope that longitudinal studies may help to elucidate how we can better prevent cognitive dysfunction in patients with AF. That being said, for now, much is still left to speculation. Separately, a significant percentage of patients who meet current recommendations for anticoagulant therapy are not treated despite not showing any contraindication.^7,8^ Prevention of dementia might thus drive appropriate utilization of this important therapy.

## Cardiac arrest

The consensus document briefly covers neurologic prognostication, memory impairment, and hypothermic protocols after cardiac arrest. The authors highlight a study by Lim et al., noting that a coma duration of less than three days after an out-of-hospital cardiac arrest (OHCA) was associated with improved late quality of life.^9^ Specifically, the data point to long-term memory impairment as the greatest risk of OHCA even in patients who recover motor function quickly. Regarding evidence-based prevention of neurologic sequelae, therapeutic hypothermia has been a mainstay since a 2003 American Heart Association (AHA) advisory statement recommended routine use; more recently, the 2010 and 2015 AHA guidelines on cardiopulmonary resuscitation reaffirmed this recommendation.^10^ A strong meta-analysis also recently showed a halved mortality rate and significantly improved neurological outcomes in patients who underwent therapeutic hypothermia after cardiac arrest.^11^ The consensus statement makes no new recommendations on this subject, as therapeutic hypothermia for patients with OHCA is the standard of care already. Again, data suggest that, despite the recommendations in place, this therapy is underutilized,^12^ demonstrating the importance of education in reinforcing clinical data—the primary goal of this expert consensus.

## Electrophysiology procedures

Catheter ablation as a nonpharmacologic rhythm-control strategy appears to lead to lower rates of stroke, death, and dementia according to one observational study.^13^ However, robust, prospective randomized data are lacking, and dementia has not been a traditional endpoint in studies evaluating catheter ablation. Nevertheless, there is some concern that periprocedural risks including subclinical cerebral ischemic events may contribute to late dementia. In a prospective study, postprocedure magnetic resonance imaging (MRI) showed new brain lesions in 43.2% of patients, 12.5% of whom appeared to show persistent scar at six months of follow-up.^14^ Despite the absence of significant neuropsychological consequences at six months, there is concern that dementia could be slowly building in such a situation and then present clinically later. Other limited studies noted evident measurable cognitive dysfunction in patients who underwent AF ablation, persisting even at 90 days.^15^ The authors of the consensus statement made the point that uninterrupted anticoagulation and perhaps more aggressive intraprocedural heparin dosing may provide some level of prevention.^1^ Additionally, in a recent 2018 study of left atrial appendage occlusion procedures, 52% of patients had MRI-detected new brain lesions postprocedure, but there was no apparent impact on cognitive testing.^16^ Currently, there are no long-term data on the impact of these procedures on cognitive functioning. The consensus statement does not provide any recommendations on this topic, but addressing the subject might drive clinical practice to the use of higher levels of anticoagulation during left-sided procedures.

## What parts of our arrhythmia management protocols could change?

More than anything, the 2018 EHRA/HRS/APHRS/LAHRS expert consensus statement was a review of the existing published literature and gaps in knowledge, without major, practice-changing recommendations. The physician-scientist could quite likely at this time push for greater utilization of anticoagulant therapy in AF as well as the standardized use of therapeutic hypothermia post-OHCA. The ablationist might use the data presented here to evaluate how they currently weigh the risks and benefits associated with higher levels of anticoagulation therapy and possibly amend their ways of doing things accordingly. However, there remains a need for large randomized clinical trials evaluating methods for preventing cognitive impairment in patients with arrhythmias. For AF patients, we may soon have data showing whether or not NOACs are superior to VKAs in preventing long-term cognitive decline. Still, as the authors note, we need a “better understanding of the mechanism and determinants of AF-related cognitive impairment/dementia” to inform future preventative strategies.

By the next consensus statement, we hope for more wide-ranging evidence for the prevention of cognitive decline in patients with arrhythmias.
